# Supplementary material for: Emergence of a Novel G4P[6] Porcine Rotavirus with Unique Sequence Duplication in NSP5 Gene in China
Source: Animals (Basel). 2024 Jun 14;14(12):1790. doi: 10.3390/ani14121790 (PMC11200575; doi:10.3390/ani14121790)
Supplement: Supplementary file 1 [file animals-14-01790-s001.zip › Supplementary Material 1-NSP5 sequence alignment.pdf]

```
Majority                 GGCTTTAAAAGCGCTACAGTGATGTCTCTCAGCATTGACGTAACGAGTCTTCCCTCAATTTCTTCTAGTATCTTTAAAAATGAATCGTCTTCTACAACGTCAACTCTTTCTGGAAAATCTATTGGTAGGAGTGAACAGTACATTTCACCAGATGCAGAAGCATTCAATAAATACATGTTGTCGAAGTCTCCAGAGGATAT
                                 10        20        30        40        50        60        70        80        90       100       110       120       130       140       150       160       170       180       190       200
JX406757 Wa.seq          .........................................G........................................................................................AC........TG.....T.....ATC............................................ 200
GU199491 Gottfried.seq   ............................................................................................C..............C.....................................................................C...................... 200
LC569890 DU2014-259.seq  ------...........................................................................................................................................................G...................................... 194
MG781041 CMH-N016-10.seq ......T.....................................A........................................................................................................................................................ 200
PP255819 S2CF.seq        ..................................................................G..................................................................................................................................... 200
JQ993328 BE2001.seq      ...........................................................................................................................................................G.....G...................................... 200

Majority                 TGGACCATCTGATTCTGCTTCAAACGATCCACTCACCAGCTTTTCGATTAGATCGAATGCAGTTAAGACAAATGCAGACGCTGGCGTGTCTATGGATTCATCGACGCAATCACGACCTTCAAGCAACGTTGGGTGCGATCAAGTGGATTTCTCCTTAACTAAAGGTATTAATGTTAGTGCTAATCTTGATTCATGTATAT
                                210       220       230       240       250       260       270       280       290       300       310       320       330       340       350       360       370       380       390       400
JX406757 Wa.seq          .........................A.............T......................................T.......................A..A....................................A.......................................G.............G... 400
GU199491 Gottfried.seq   ...........................C............................................................................................................................................................................ 400
LC569890 DU2014-259.seq  ...........................C.............................................................................A.................T........A.........................................A......................... 394
MG781041 CMH-N016-10.seq .........................................................................................................A..................................................G........................................... 400
PP255819 S2CF.seq        .......................................T..............................................................A.........................................................................A..................C.... 400
JQ993328 BE2001.seq      ......................................................................................................................................A.................................C.......A..................C.... 400

Majority                 CAATTTCAACTGATCATAAAAAGGAGAAATCCAAGAAAGATAAAAGTAGGAAACACTACCCGAGAATTGAAGCAGATTCTGATTCTGAAGATTATGTTTTAGATGATTCAGATAGTGATGACGGTAAATGTAAGAATTGTAAATATAAGAAAAAGTATTTCGCACTAAGAATGAGGATGAAGCAAGTCGCAATGCAATTG
                                410       420       430       440       450       460       470       480       490       500       510       520       530       540       550       560       570       580       590       600
JX406757 Wa.seq          ...........A.C.................T..A..G.......................A....................C.A.........C.....G...............................................A..G..A.....T....................A.................. 600
GU199491 Gottfried.seq   ....C..G................................................................................................................................................................................................ 600
LC569890 DU2014-259.seq  ....C..............G..........................................................................C............................................C............................................................ 594
MG781041 CMH-N016-10.seq ................C............................A......................................................G................................................................................................... 600
PP255819 S2CF.seq        ....A..........................A..A.............A............A...............................................G.......................................................................................... 600
JQ993328 BE2001.seq      ....A..............G............................................G.............................C.................................................................T....................................... 600

Majority                 ATCGAAGATTTGTAATGTCAACC------------TGA----GGGCGCACTAGGGAGCTCCCCACTCCCGTTTTGTGACCXXXXXXXXXXXXXXXXXXXXXXXXXXXXXXXXXXXXXXXXXXXXXXXXXXXXXXXXXXXXXXXXXXXXXXXXXXXXXXXXXXXXXXXXXXXXXXXXXXXXXXXXXXXXXXXXXXXXXXXX
                                610       620       630       640       650       660       670       680       690       700       710       720       730       740       750       760       770       780       790       800
JX406757 Wa.seq          ..A................G...------------...----..A...................................                                                                                                                        664
GU199491 Gottfried.seq   .......................------------...----.A..A.................................                                                                                                                        664
LC569890 DU2014-259.seq  .......................------------...----.A..A.................................                                                                                                                        658
MG781041 CMH-N016-10.seq .......................------------...----.A..A.................................                                                                                                                        664
PP255819 S2CF.seq        .....................T.ACAACCTTCAAGCA.CGTT...T..GA.C.A.T.GATTT.T..TTGAC.AAAG.TATTAACGTTAATGCTAATCTTGATTTACGTATATCAATATCAACTAATCATAAAAAGGAGAAATTCAAAAAAGATAAAAGTAGGAAACACTACCCAAGAATTGAAGCAGATTCTGATTTTGA 800
JQ993328 BE2001.seq      ....................GT.------------...CGTT...T..GA.T.A.C.GATTT.T.CTTAAC.AAAG.GATTAATATTAATGTTAATCTTAATTCACGTATATCAATATCAACTAATTACAAGATGGAGAAATCCAAGAAAGATAAAAGTAGAAAACACTATTCGAGGATTGAAGCAGATTCTGATTCTGG 788

Majority                 XXXXXXXXXXXXXXXXXXXXXXXXXXXXXXXXXXXXXXXXXXXXXXXXXXXXXXXXXXXXXXXXXXXXXXXXXXXXXXXXXXXXXXXXXXXXXXXXXXXXXXXXXXXXXXXXXXXXXXXXXXXXXXXXXXXXXXXXXXXXXXXXXXXXXXXXXXXXXXXXXXXXXXXXX
                                810       820       830       840       850       860       870       880       890       900       910       920       930       940       950       960       970
JX406757 Wa.seq                                                                                                                                                                                                              664
GU199491 Gottfried.seq                                                                                                                                                                                                       664
LC569890 DU2014-259.seq                                                                                                                                                                                                      658
MG781041 CMH-N016-10.seq                                                                                                                                                                                                     664
PP255819 S2CF.seq        AGATTATACTTTAGATGGTTTAGGTAATGATGACGGTAAATGTAAAA-TTGTAAATATAAGAAAGAGTATTTCACACTAAGAATGAGAATGAAGCAAATCGCAATGCAATTGATCGAAGATTCGTGATGTCAACCTGAGAGCACACTAGGGAGCTCCCCACTC                                         962
JQ993328 BE2001.seq      TGATTATTTTTCAGATGATTCACATAGTGATGAAAGTAAATGTAAGAGTTGTAAATATAAGAAGAAGCATTTTGCACTAAGAATGAGGATGAATCAAGTCGCAAAGCAGTTGATCGAAGATTTGTAATGTCGACCTGAGAGCACACTAGGGAGCTCCCCACTCCCGTTTTGTGACC                            964
```

Decoration 'Decoration #1': Hide (as '.') residues that match the Consensus exactly.

Decoration 'Decoration #2': Hide (as '.') residues that match the Consensus exactly.
