# Supplementary material for: Emergence of a Novel G4P[6] Porcine Rotavirus with Unique Sequence Duplication in NSP5 Gene in China
Source: Animals (Basel). 2024 Jun 14;14(12):1790. doi: 10.3390/ani14121790 (PMC11200575; doi:10.3390/ani14121790)
Supplement: Supplementary file 1 [file animals-14-01790-s001.zip › Supplementary Material 3-duplication region.pdf]

Alignment Report of Untitled ClustalW (Slow/Accurate, IUB)

Sunday, April 14, 2024 12:01 AM

```
Majority            GGCTTTAAAAGCGCTACAGTGATGTCTCTCAGCATTGACGTAACGAGTCTTCCCTCAATTTCTTCTGGTATCTTTAAAAATGAATCGTCTTCTACAACGT
                             10        20        30        40        50        60        70        80        90       100
344 PP255819 S2CF.seq  ---------------------------------------------------------------------------------------------------- 0
618 PP255819 S2CF.seq  .................................................................................................... 100
962 PP255819 S2CF.seq  .................................................................................................... 100

Majority            CAACTCTTTCTGGAAAATCTATTGGTAGGAGTGAACAGTACATTTCACCAGATGCAGAAGCATTCAATAAATACATGTTGTCGAAGTCTCCAGAGGATAT
                            110       120       130       140       150       160       170       180       190       200
344 PP255819 S2CF.seq  ---------------------------------------------------------------------------------------------------- 0
618 PP255819 S2CF.seq  .................................................................................................... 200
962 PP255819 S2CF.seq  .................................................................................................... 200

Majority            TGGACCATCTGATTCTGCTTCAAACGATCCACTCACCAGTTTTTCGATTAGATCGAATGCAGTTAAGACAAATGCAGACGCTGGCGTGTCTATGGATTCA
                            210       220       230       240       250       260       270       280       290       300
344 PP255819 S2CF.seq  ---------------------------------------------------------------------------------------------------- 0
618 PP255819 S2CF.seq  .................................................................................................... 300
962 PP255819 S2CF.seq  .................................................................................................... 300

Majority            TCAACGCAATCACGACCTTCAAGCAACGTTGGGTGCGATCAAGTGGATTTCTCCTTAACTAAAGGTATTAATGTTAATGCTAATCTTGATTCATGCATAT
                            310       320       330       340       350       360       370       380       390       400
344 PP255819 S2CF.seq  -----........A..........................G............T..G..............C...................T.C.T.... 94
618 PP255819 S2CF.seq  .................................................................................................... 400
962 PP255819 S2CF.seq  .................................................................................................... 400

Majority            CAATATCAACTGATCATAAAAAGGAGAAATCAAAAAAAGATAAAAGTAAGAAACACTACCCAAGAATTGAAGCAGATTCTGATTCTGAAGATTATGTTTT
                            410       420       430       440       450       460       470       480       490       500
344 PP255819 S2CF.seq  ...........A..................TC................G...................................T..........AC... 194
618 PP255819 S2CF.seq  .................................................................................................... 500
962 PP255819 S2CF.seq  .................................................................................................... 500

Majority            AGATGATTCGGATAGTGATGACGGTAAATGTAAGAATTGTAAATATAAGAAAAAGTATTTCGCACTAAGAATGAGGATGAAGCAAGTCGCAATGCAATTG
                            510       520       530       540       550       560       570       580       590       600
344 PP255819 S2CF.seq  .....G..TA.G..A..................-..................G........A.............A.........A.............. 293
618 PP255819 S2CF.seq  .................................................................................................... 600
962 PP255819 S2CF.seq  .................................................................................................... 600
```

```
Majority                 ATCGAAGATTTGTAATGTCAAXCXXAXXXXXCAXXXXXXXXXXXXXXXXXXXXXXXXXXXXXXXXXXXXXXXXXXXXXXXXXXXXXXXXXXXXXXXXXXX

                                  610       620       630       640       650       660       670       680       690       700

344 PP255819 S2CF.seq    .........C..G.......C.TG.GAGCA..CTAGGGAGCTCCCCACTC                                                  344
618 PP255819 S2CF.seq    ..................                                                                                  618
962 PP255819 S2CF.seq    ....................T.AC.ACCTT..AGCAACGTTGGGTGCGATCGAGTGGATTTCTCTTTGACTAAAGGTATTAACGTTAATGCTAATCTTG 700

Majority                 XXXXXXXXXXXXXXXXXXXXXXXXXXXXXXXXXXXXXXXXXXXXXXXXXXXXXXXXXXXXXXXXXXXXXXXXXXXXXXXXXXXXXXXXXXXXXXXXXXXX

                                  710       720       730       740       750       760       770       780       790       800

344 PP255819 S2CF.seq                                                                                                        344
618 PP255819 S2CF.seq                                                                                                        618
962 PP255819 S2CF.seq    ATTTACGTATATCAATATCAACTAATCATAAAAAGGAGAAATTCAAAAAAGATAAAAGTAGGAAACACTACCCAAGAATTGAAGCAGATTCTGATTTTGA 800

Majority                 XXXXXXXXXXXXXXXXXXXXXXXXXXXXXXXXXXXXXXXXXXXXXXXXXXXXXXXXXXXXXXXXXXXXXXXXXXXXXXXXXXXXXXXXXXXXXXXXXXXX

                                  810       820       830       840       850       860       870       880       890       900

344 PP255819 S2CF.seq                                                                                                        344
618 PP255819 S2CF.seq                                                                                                        618
962 PP255819 S2CF.seq    AGATTATACTTTAGATGGTTTAGGTAATGATGACGGTAAATGTAAAATTGTAAATATAAGAAAGAGTATTTCACACTAAGAATGAGAATGAAGCAAATCG 900

Majority                 XXXXXXXXXXXXXXXXXXXXXXXXXXXXXXXXXXXXXXXXXXXXXXXXXXXXXXXXXXXXXX

                                  910       920       930       940       950       960

344 PP255819 S2CF.seq                                                                                                        344
618 PP255819 S2CF.seq                                                                                                        618
962 PP255819 S2CF.seq    CAATGCAATTGATCGAAGATTCGTGATGTCAACCTGAGAGCACACTAGGGAGCTCCCCACTC                                      962
```

Decoration 'Decoration #1': Hide (as '.') residues that match the Consensus exactly.
