# Supplementary material for: Emergence of a Novel G4P[6] Porcine Rotavirus with Unique Sequence Duplication in NSP5 Gene in China
Source: Animals (Basel). 2024 Jun 14;14(12):1790. doi: 10.3390/ani14121790 (PMC11200575; doi:10.3390/ani14121790)
Supplement: Supplementary file 1 [file animals-14-01790-s001.zip › Supplementary Material 4-Phylogenetic trees of VP6, VP1,VP2,VP3, NSP1, NSP2, NSP3, NSP4.pdf]

Supplementary Meterial S4. The phylogenetic trees of VP6, VP1, VP2, VP3, NSP1, NSP2, NSP3, NSP4.

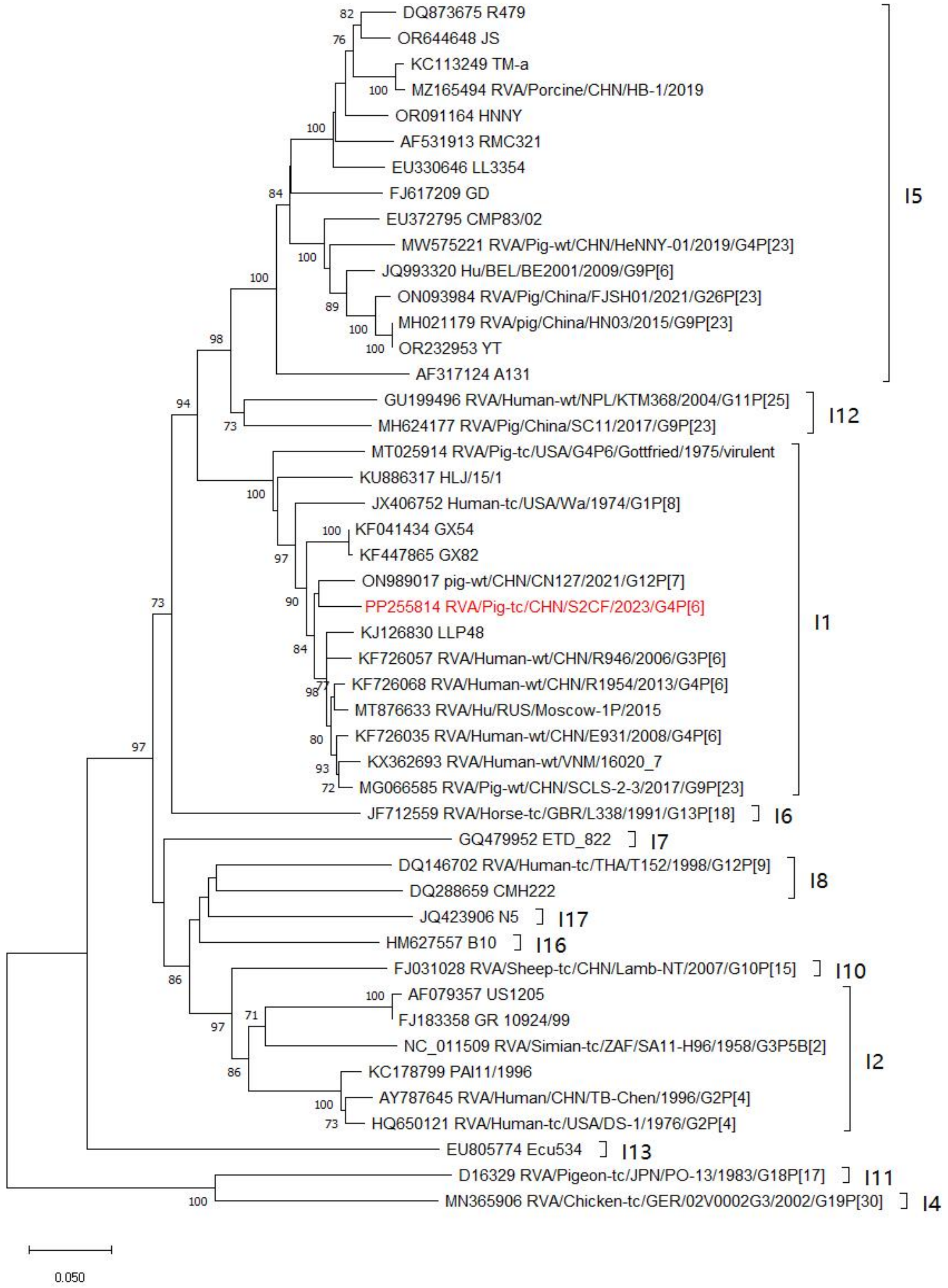


**Figure S1.** Phylogenetic tree based on VP6 was constructed using neighbor-joining method with 1,000 bootstrap replicates under the K2 + G model. The bootstrap values over 70% are indicated at each branch node represents substitutions per nucleotide site. The S2CF strain isolated in this study was marked in red. Abbreviation: K2= Kimura 2-parameter, G= Gamme sites.

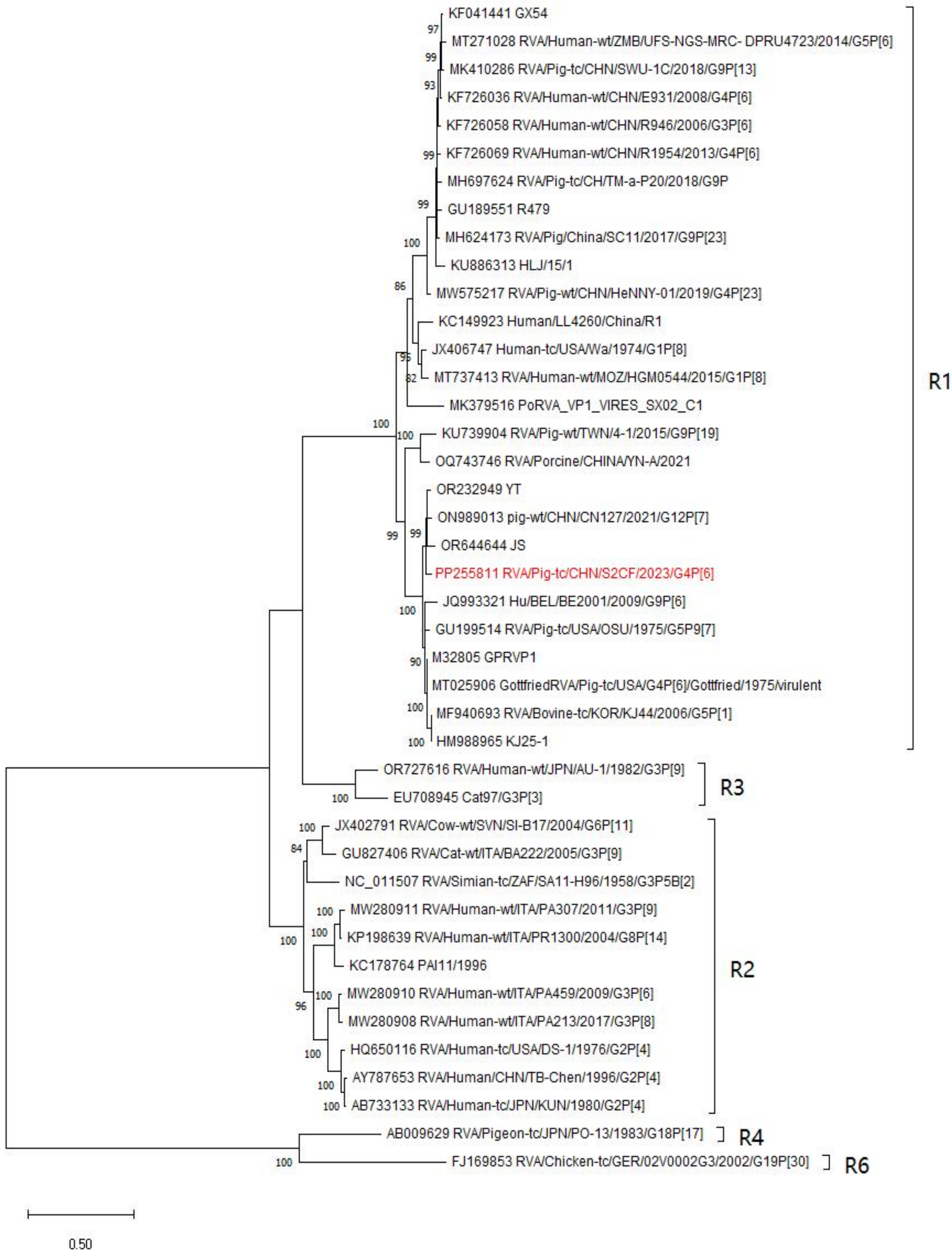


**Figure S2.** Maximum-likelihood phylogenetic tree based on the VP1 was finished using genetic distances calculated with 1,000 bootstrap replicates under the GTR + G + I model. Bootstrap values ⩾ 70%. The S2CF strain isolated in this study is marked with red. Abbreviation: GTR= General Time Reversible, G= Gamme sites, I= Invariant sites.

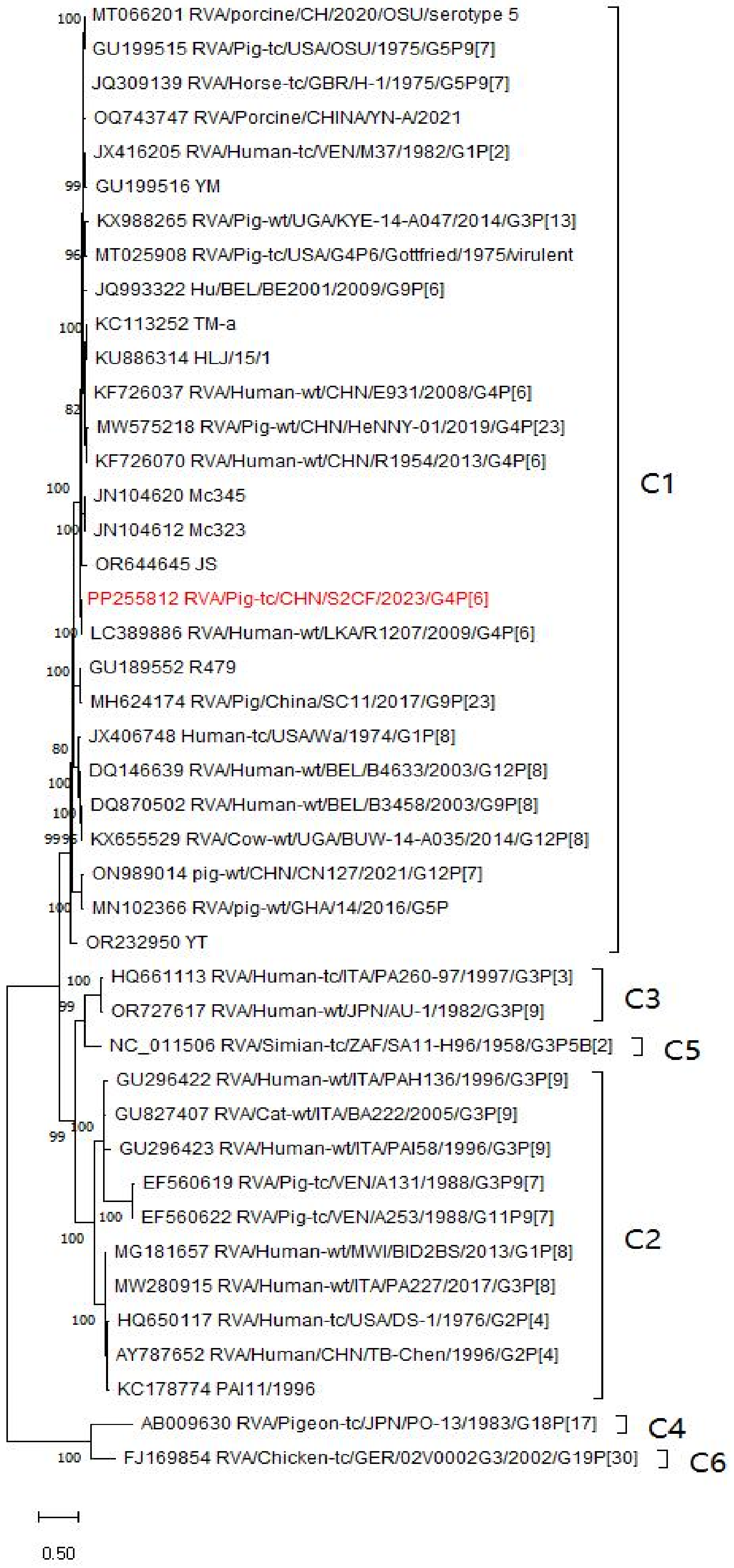


**Figure S3.** Phylogenetic tree of VP2 was finished using genetic distances calculated by maximum likelihood with 1,000 bootstrap replicates under the GTR + G + I model. Node labels represents bootstrap values ⩾ 70% are shown. The S2CF strain isolated in this study is marked with red.

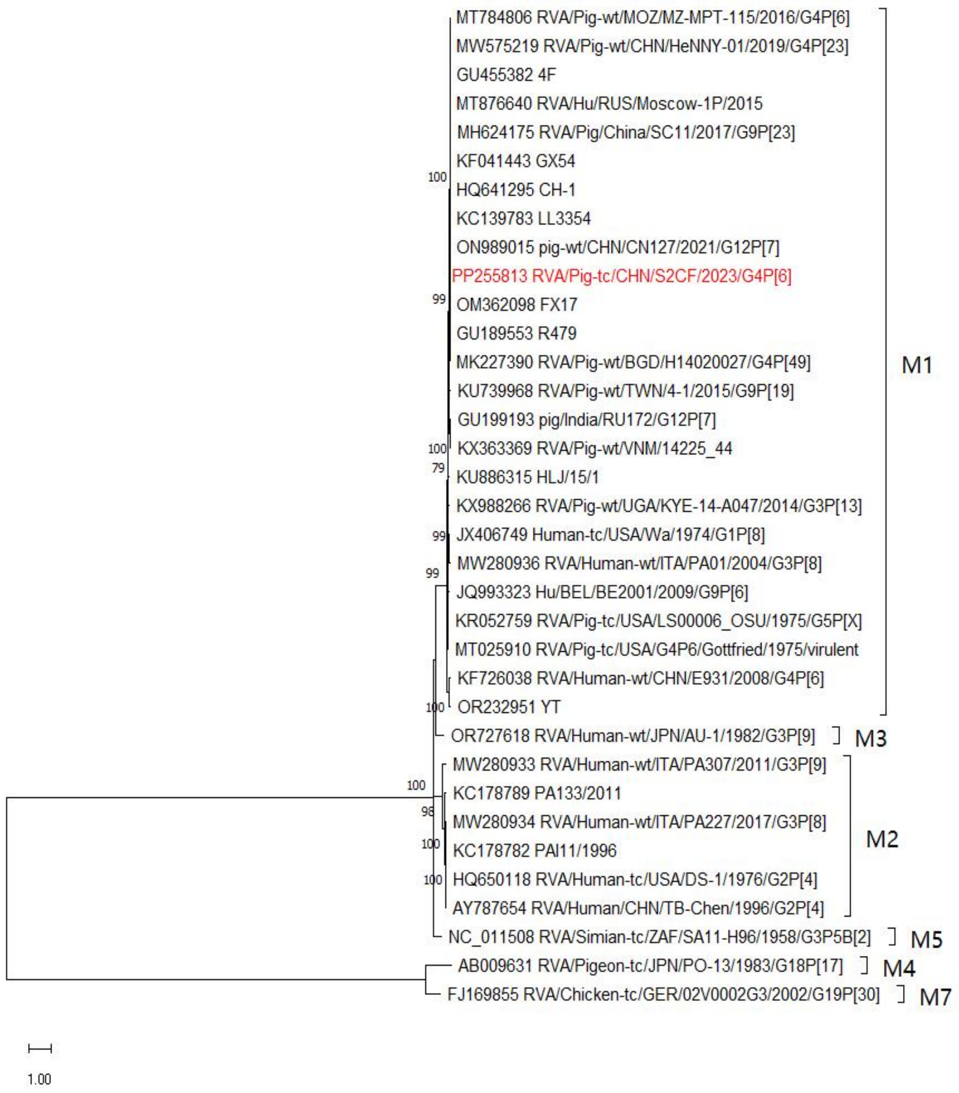


**Figure S4.** Phylogenetic tree of VP3 was finished using genetic distances calculated by maximum likelihood with 1,000 bootstrap replicates under the GTR + G + I model. Node labels represents bootstrap values ≥ 70% are shown. The S2CF strain isolated in this study is marked with red.

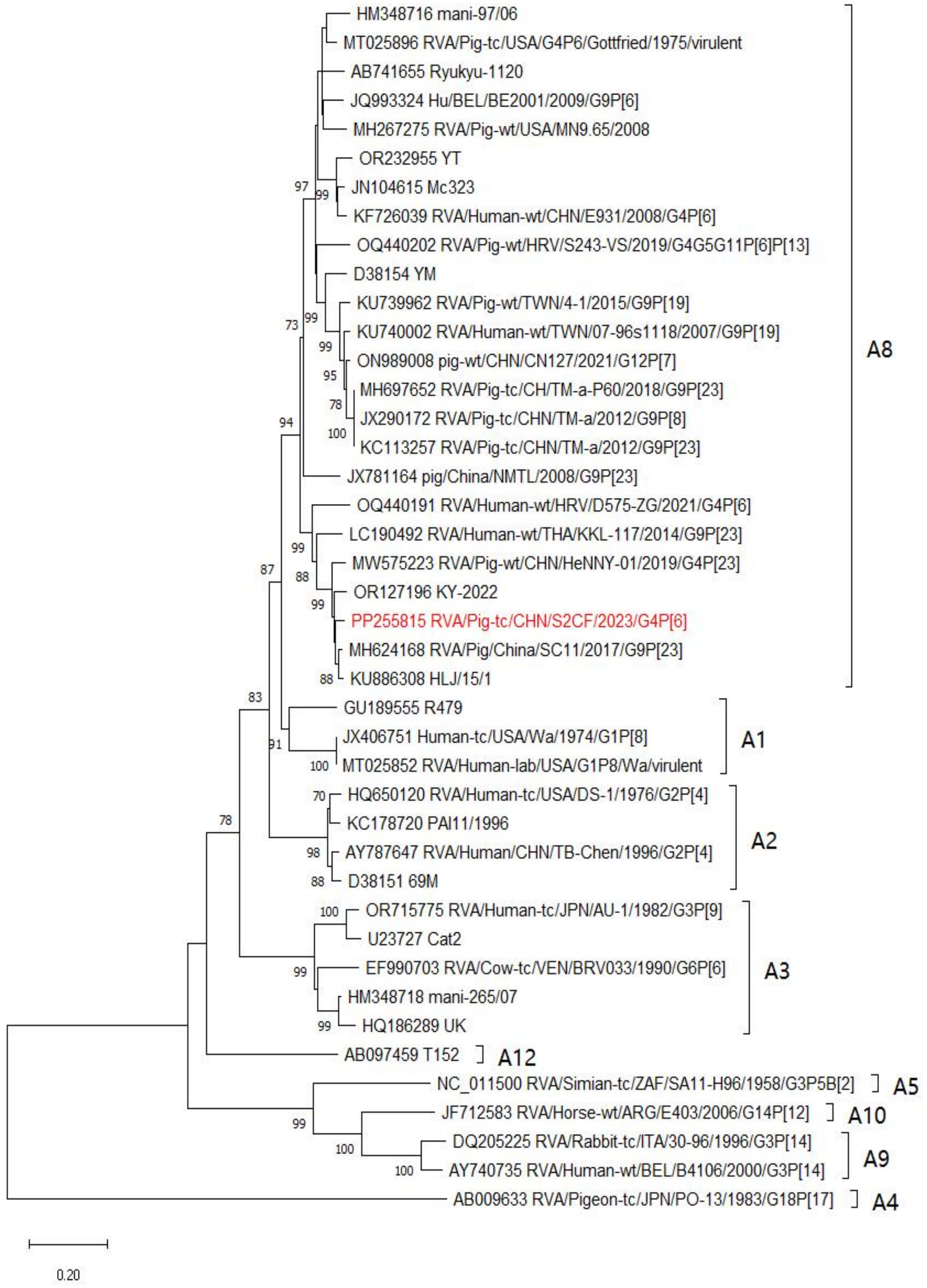


**Figure S5.** Phylogenetic tree based on NSP1 was constructed using neighbor-joining method with 1,000 bootstrap replicates. The model was K2+G. The bootstrap values ≥ 70% are indicated at each branch node represents substitutions per nucleotide site. The S2CF strain isolated in this study was marked in red.

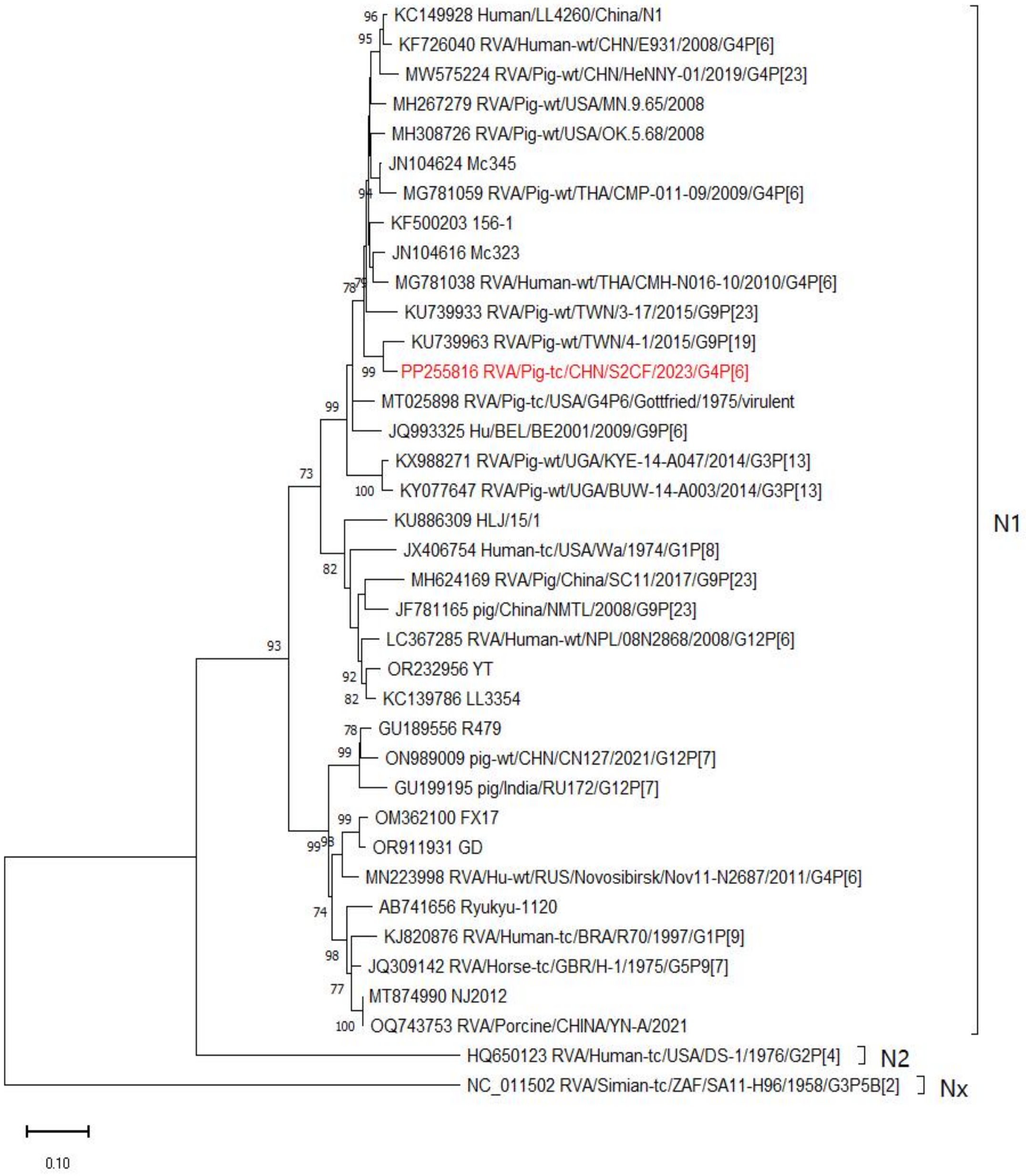


**Figure S6.** The phylogenetic tree constructed from the nucleotide of NSP2 based on the maximum likelihood method under the T92 + G model and boot-strapped with 1000 repetitions. The bootstrap values ⩾ 70%r are indicated at each branch node represents substitutions per nucleotide site. The S2CF strain isolated in this study was marked in red.

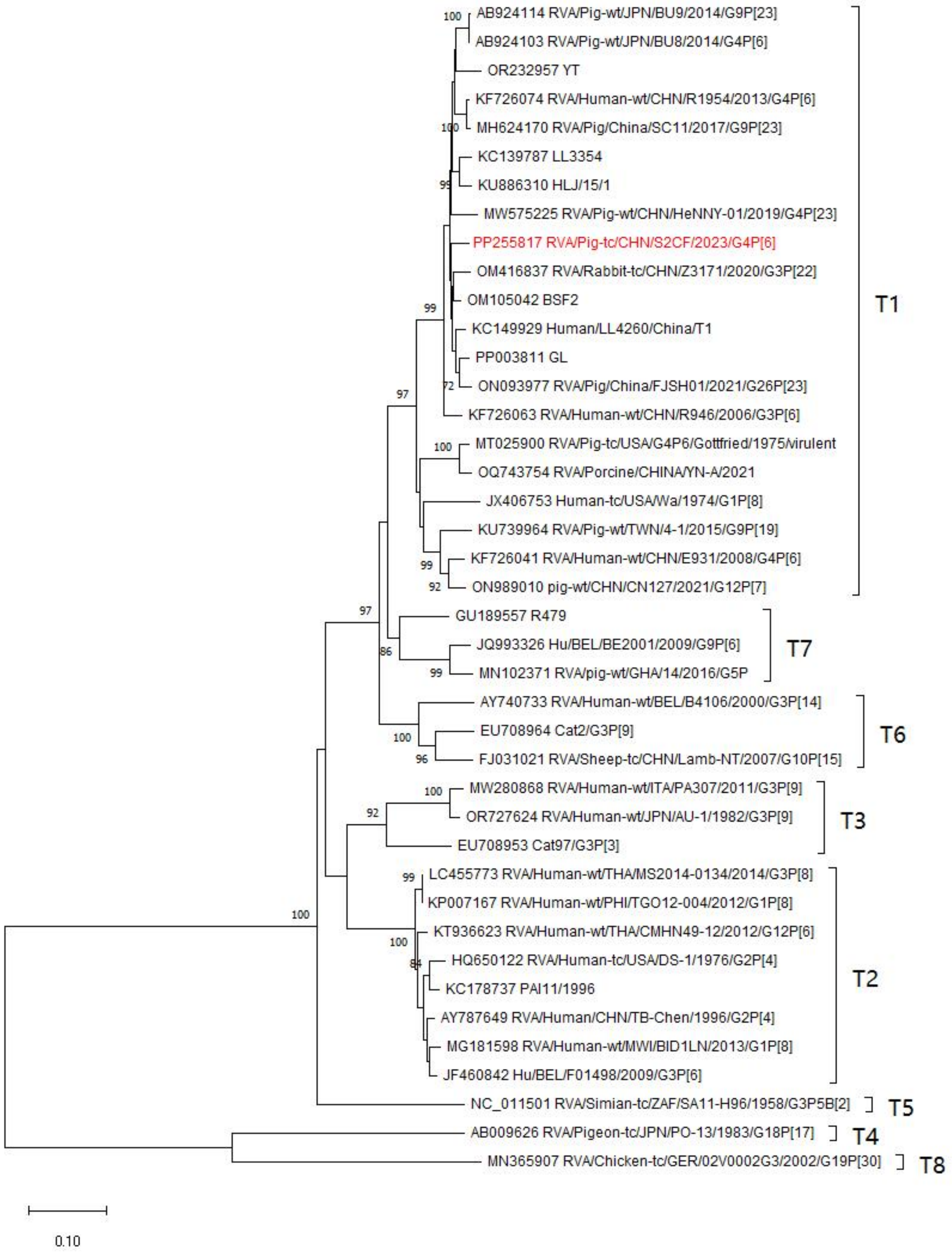


**Figure S7.** Phylogenetic tree of NSP3 was constructed using neighbor-joining method with 1,000 bootstrap replicates. The bootstrap values ≥ 70% are indicated at each branch node represents substitutions per nucleotide site. The S2CF strain isolated in this study was marked in red.

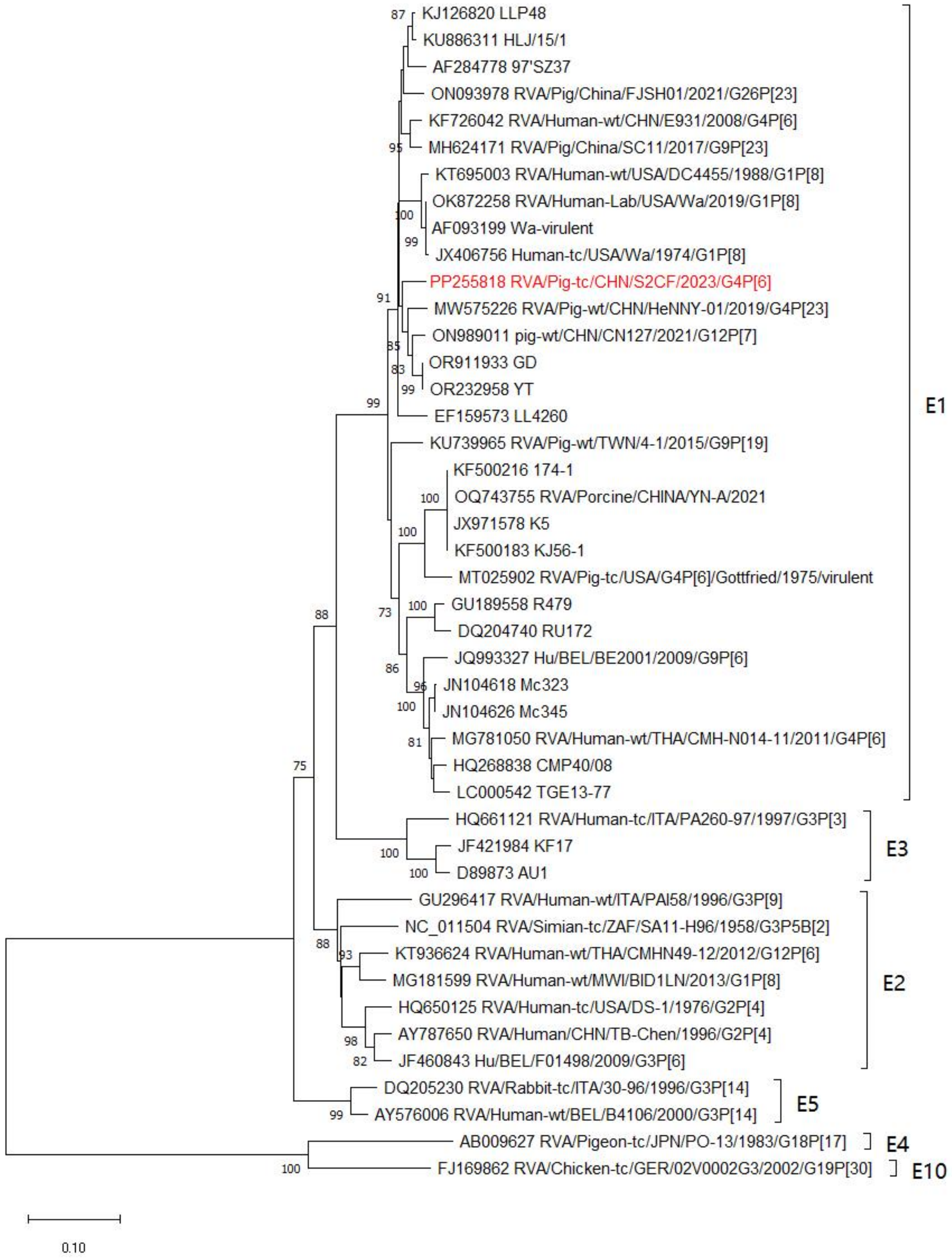


**Figure S8.** Phylogenetic tree of NSP4 was constructed using neighbor-joining method with 1,000 bootstrap replicates. The bootstrap values ≥ 70% are indicated at each branch node represents substitutions per nucleotide site. The S2CF strain isolated in this study was marked in red.
