# Supplementary figures and images for: Emergence of a Novel G4P[6] Porcine Rotavirus with Unique Sequence Duplication in NSP5 Gene in China
Source: Animals (Basel). 2024 Jun 14;14(12):1790. doi: 10.3390/ani14121790 (PMC11200575; doi:10.3390/ani14121790)

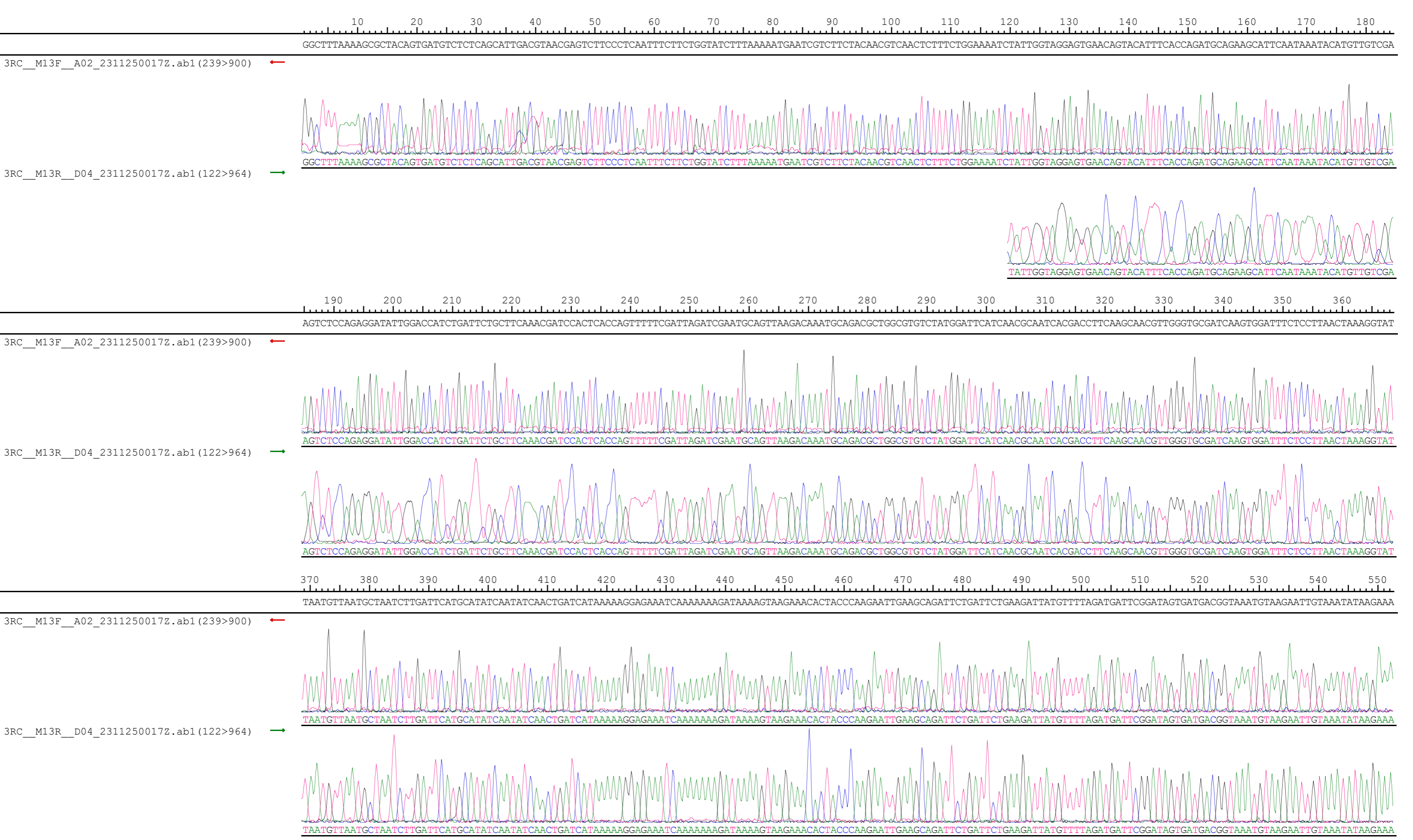

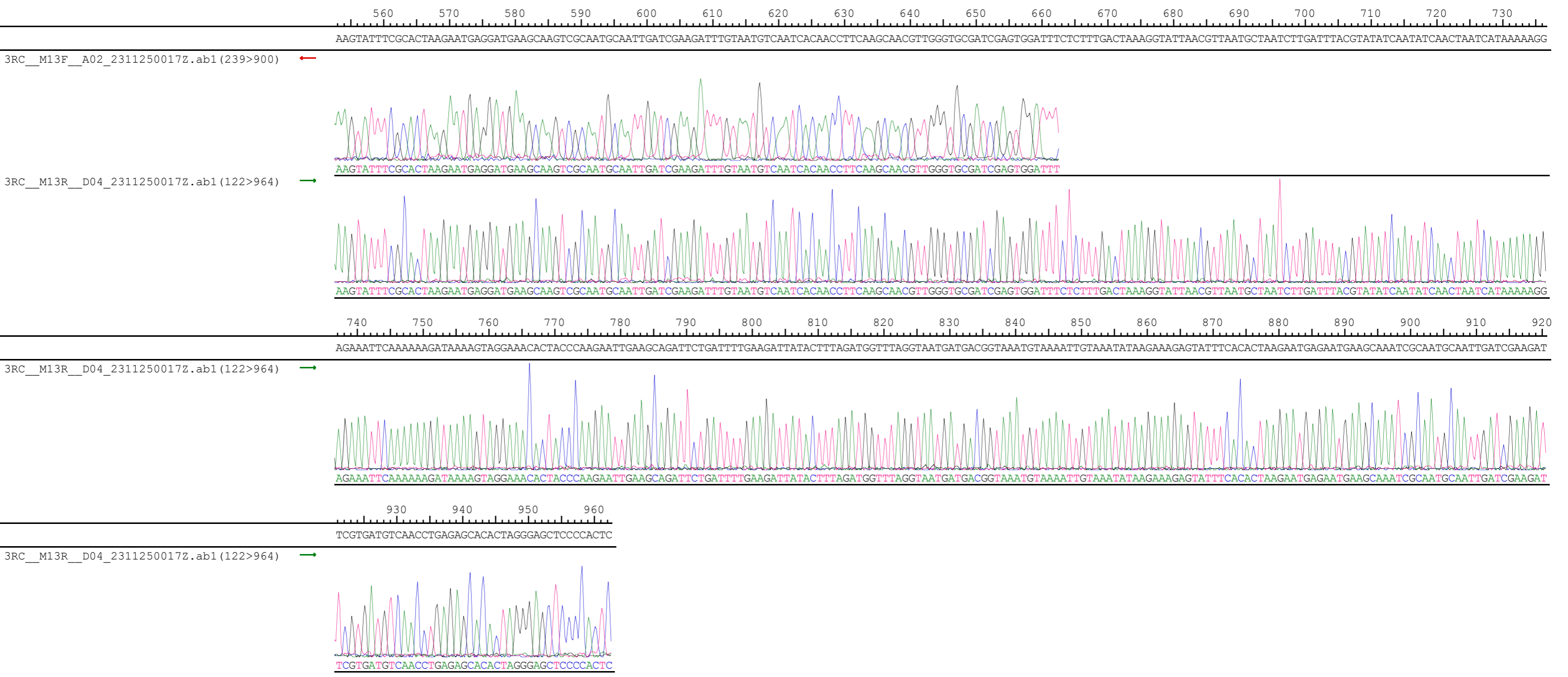

Supplement: Supplementary file 1 [file animals-14-01790-s001.zip › Supplementary Material 2-Sanger sequencing region (619-962).pdf]
